# Solid oxide fuel cell simulation model tuning using operating conditions dependent optimization techniques

**DOI:** 10.1038/s41598-025-34342-3

**Published:** 2026-01-28

**Authors:** Haya Hesham, Mohamed Abdel Rahman, Ghada Bassioni, Rania A. Swief, Mohamed Ezzat, Sherif Helmy, Nourhan M. Elbehairy

**Affiliations:** 1 Electrical Power and Machines Department, Faculty of Engineering, Ain Shams University, Cairo, Egypt; 2 Engineering Physics and Mathematics Department, Faculty of Engineering, Ain Shams University, Cairo, Egypt; 3 Electrical Power and Energy Department, Military Technical College, Cairo, Egypt; 4 Energy and Renewable Energy Department, Faculty of Engineering and Technology, Egyptian Chinese University, Cairo, Egypt

**Keywords:** Simulation, Model, SOFC, V-I polarization curve, Operating conditions, WaOA, Energy science and technology, Engineering, Mathematics and computing

## Abstract

This paper discusses the mathematical model and simulation of Solid Oxide Fuel Cell (SOFC), where the conventional and reversible SOFC are studied. Their performance is studied at the steady state to get the V-I polarization curves, as well as in case of changing the electric current drawn from the cell to get the change in voltage over time. Unlike most existing studies, the focus is on the time needed by the cell to reach voltage stability after a change, in preparation for studying the cell integration into an electrical network, and all of these studies are carried out when changing the cell operating conditions such as temperatures and the flow rate of reactive gases. Then optimization techniques are used, such as Walrus Optimization Algorithm (WaOA), Secretary Bird Optimization Algorithm (SBOA), Chaos Game Optimization (CGO) and Teaching–Learning Based Optimization (TLBO) to increase model accuracy and make its results closer to the published laboratory results. The results demonstrate that WaOA achieves the lowest modeling error among the investigated optimization techniques and provides the most accurate model calibration. The proposed WaOA-based tuning reduces the modeling error by more than five orders of magnitude compared to curve-fitting approaches reported in the literature, and is further employed to optimize reactant flow rates, resulting in an output power increase of approximately 6% compared to nominal operating conditions.

## Introduction

**Fig. 1:**
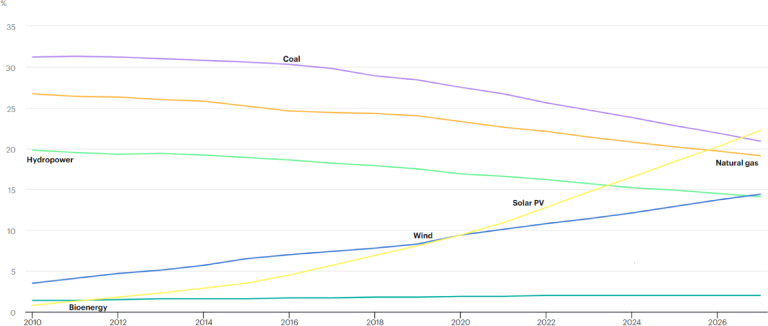
Technology-specific share of worldwide cumulative electricity capacity, 2010–2027^3^.

The global average temperature has risen by approximately 1.1 °C over the pre-industrial age due to greenhouse gas emissions. That is known as the global warming crisis. To face this worrying trend and guarantee that the rise in global temperature is kept to 1.5 °C over pre-industrial age, net zero emissions by 2050 and a 45% reduction in emissions by 2030 must be achieved^1^. The energy sector is a primary cause of the urgency surrounding this issue, accounting for around 75% of current greenhouse gas emissions^2^. The best method to address this issue is to stop using gas, oil, and coal-fired electricity, emitting pollution. Rather, as a sustainable and eco-friendly substitute, renewable energy sources must be given the highest priority and financial support. Figure 1 shows how the world is shifting towards a higher dependency on solar power, wind power, and other renewable energy resources in comparison to conventional methods of generating electricity^3^. EIA statistics indicate that the global capacity for electricity generation in 2023 is 9080 GW, with solar and wind power constituting 24% of this total^4^.

Solar and wind energy are two of the most widely used types of renewable energy. But these energy sources have a serious drawback. They are inherently intermittent. The amount of energy generated from these sources depends on climatic conditions such as sun irradiance and wind speed, which leads to more interest in energy storage systems, one of which is hydrogen energy. Hydrogen presents a viable alternative energy source that can address the instability problems related to solar and wind power since it can provide energy constantly and reliably.

**Fig. 2:**
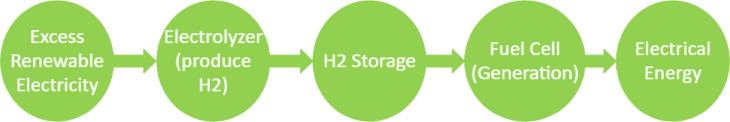
Three-stage process of hydrogen energy.

The three stages of hydrogen energy, production, storage, and electricity generation are how this promising renewable energy source works. The fundamental idea behind this technology is hydrogen production using excess renewable energy. An electrolyser running on renewable energy is used to do this^5^. When hydrogen is produced, it may be stored and then used by a fuel cell to generate electricity, particularly when demand from consumers exceeds supply from renewable sources^6^. This process is indicated in Fig. 2.

Fuel cells are one of the most common energy storage systems now, as they have many advantages, such as high efficiency, lower pollution levels, almost zero noise levels, and different types that suit all applications. Among these technologies, Proton Exchange Membrane Fuel Cells (PEMFCs) and Solid Oxide Fuel Cells (SOFCs) are the most widely studied and deployed. PEMFCs are commonly favored for low-temperature and fast-response applications, particularly in transportation systems^7–9^, whereas SOFCs offer key advantages for stationary power generation, including fuel flexibility and tolerance to fuel impurities, which solves problems related to hydrogen storage and hydrogen purification^10^. SOFC can also be combined with heat engines to form a hybrid power generation system, which increases the efficiency of power generation^11^.

The static model of an anode-supported tubular SOFC is presented in^12^, and the simulation results of the SOFC model are compared with actual data from the lab to validate the model. The dynamic and static model of a SOFC, which considers the effect of electrochemical, mass flow, and thermal, is presented in^13^. However, temperature dynamics are not taken into consideration in this model. Dynamic models of SOFCs are proposed in^14^ and^15^ to analyze fuel cells and power system performance. While species dynamics is included in these models, temperature dynamics is not taken into account. In^16^, a more comprehensive mathematical model of an SOFC is carried out. It estimates the composition and particle size of a cathode microstructure SOFC model parameters. In^17^, a model is proposed to indicate the limitations of the empirical presumptions drawn from physical process observation and measurement. A combination of physical modeling and data-driven simulation has been studied to represent multi-physics phenomena in SOFC^18^. A dynamic fuel cell model that employs a similar approach to that in^14^, is set in^19^. In^20^, the transient response of a SOFC has been modeled using a multi-input neural network for accurate and time-efficient simulation. A state-of-the-art multi-physics model has integrated thermal, mechanical and electronic aspects while improving cell performance in^21^. Reference^22^ presents a three-dimensional model with dynamic analysis and studies the effect of carbon deposition on the cell performance. The impact of polarization losses on an anode-supported SOFC is the main topic of reference^23^. Both the thermal and electrochemical features of the chemical processes occurring inside the fuel-cell stack are included in its model. In^24^, A single cell is split into small control volumes (CVs) in a dynamic model of the SOFC. The fuel cell model presented in reference^25^ is based on empirical equations obtained from real fuel cell lab data. A mathematical model of a planar SOFC is shown in reference^26^, with a focus on how temperature variations affect output voltage response. In^27^, the dynamic behavior of a stand-alone SOFC resulting from a change in load is studied. It shows how closely the influence of temperature dynamics is connected to the relaxation period of the output voltage. When the operational conditions, such as inlet oxygen flow rate, inlet hydrogen temperature, and inlet oxygen temperature, are changed, the static and dynamic performance of the tubular SOFC are compared in^28^. Modeling of reversible SOFC (rSOFC) is presented in^29^ with the focus on the internal structure of the cell. In reference^30^, a 5kW rSOFC system is designed and constructed. A grid-scale (1MW) rSOFC is studied in^31^ during its connection to a hydrogen pipeline. In^32^, SOFC is used to connect between the electrical grid and gas network, so the cell is studied at fuel cell mode and electroyser mode. A rSOFC is used in electroyser mode for hydrogen production with the focus on system efficiency^33^. The dynamic properties of the rSOFC in various operational modes and switching processes are analyzed in references^34–36^. In^37^, the critical operational factors influencing the rSOFC stack’s dynamic behavior during mode switching are covered. In addition to the well-established foundational models that remain widely adopted as benchmarks in recent studies, a growing body of recent literature has focused on advanced dynamic modeling, optimization-based techniques^40–46^, and system-level integration of SOFC and rSOFC technologies, reflecting the ongoing research interest and the relevance of the proposed work.

Despite the significant progress in SOFC and rSOFC modeling, recent studies largely focus on improving physical and data-driven representations and analyzing transient behavior under specific operating conditions. However, the systematic use of modern optimization techniques as a unified framework for SOFC model tuning remains limited in the published literature, particularly when linking model calibration to dynamic response characteristics, such as the voltage stabilization time and operating-condition-dependent performance. In addition, the use of optimization techniques to adjust reactant flow rates and to explicitly quantify their impact on the output power has received limited attention in the literature. This gap motivates the comprehensive optimization-based framework adopted in the present study.

This study can be divided into three main parts, all based on the modeling of the fuel cell.

The first part focuses on deriving the relationship between voltage and current, which is commonly referred to as the polarization curve, for both the SOFC and the rSOFC, which operates in two modes: fuel cell mode and electrolyser mode.

The second part investigates the time required for the cell voltage to reach the steady state following a change in the drawn current. This also applies to both the SOFC and the rSOFC. This section differs from previous works in the literature, which primarily focus on the internal changes occurring within the SOFC itself during current variation, such as the temperature gradient along the cell.

The third part addresses modeling optimization and is further divided into two sections: the first deals with changing parameters within the model to enhance its accuracy and make the simulation results closer to the laboratory results reported in the previously published works. This is achieved by comparing the V-I polarization curve generated by the model with that obtained from experimental measurements. Several optimization techniques are employed to determine the optimal parameter values, and their results are compared to identify the most accurate outcome. Although optimization techniques have not been widely applied in the context of fuel cell modeling in the literature, they yield satisfactory results in this case. The second section focuses on minimizing the time required for the cell voltage to stabilize after a change in the drawn current. This is approached by altering the operating conditions of the cell, such as the reactants flow rates, operating temperature, and the magnitude of current variation. Additionally, this section investigates the effect of varying the reactants flow rates on the amount of output power that can be obtained from the cell, which is a topic not previously addressed in published works. This analysis is conducted in light of the observation that varying the flow rates of both oxygen and hydrogen has produced similar results in terms of the voltage stabilization time.

The structure of this paper is as follows: The hydrogen energy process is introduced in Section I. Section II covers the theory of operation for the fuel cell and electrolyser, as well as the chemical reactions that take place during their operation. The SOFC and the associated equations required to build the models are also covered in Section II. Section III covers the methodology of the paper. It discusses the relationship between voltage and current in conventional and reversible SOFCs. It also presents the dynamic response and how the SOFC voltage varies when the current changes. Lastly, Section IV represents the conclusion of the work.

## Operation and modelling equations

One of the most interesting studies in hydrogen energy is water electrolysis in producing hydrogen and fuel cells in generating electricity. This will be discussed in this section, as it will include chemical reactions and the theory of operation of electroyser and fuel cell, especially SOFC and the mathematical equations used in constructing its model.

A. Electrolyser

**Fig. 3:**
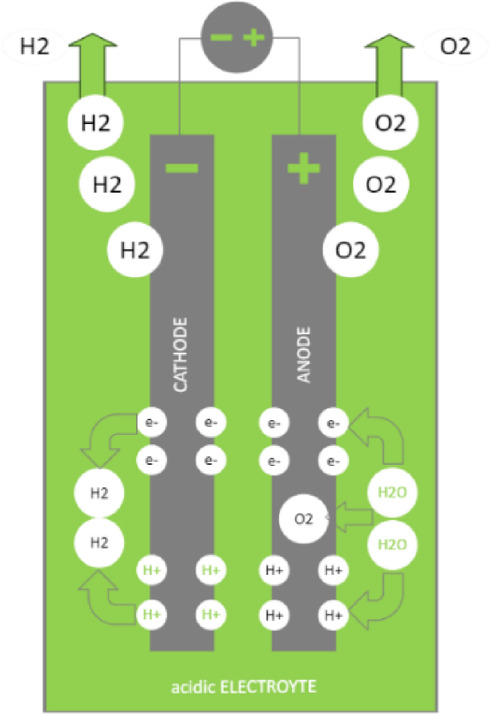
Electrolyser.

The basic idea behind an electrolyser is not very complicated. It uses two graphite electrodes—the anode and the cathode, respectively—that are dipped in an acidic electrolyte, as shown in Fig. 3. Usually, this electrolyte is a solution of ${\mathrm{H}}_{2}\mathrm{O}$ with HCL, ${\mathrm{H}}_{2}{\mathrm{SO}}_{4}$, or another acid. Pure H_2_O is a poor electrical conductor; thus, adding the acid is essential. When the acid is present, the H_2_O separates into H^+^ and OH^-^ ions^6^.

1 $$2{\mathrm{H}}_{2}\text{O }\to 2{\mathrm{H}}^{+}+2{\mathrm{OH}}^{-}$$

H^+^ ions are pulled to the anode, and OH^-^ ions are attracted to the cathode when a DC voltage is applied on the anode and the cathode. The DC voltage is theoretically 1.23 V, but practically, it is higher than this value. There is a greater tendency for electron loss in OH^-^ ions.

2 $${\mathrm{OH}}^{-} \to \mathrm{OH} + {\mathrm{e}}^{-}$$

After that, the electron moves to the cathode through the external electrical circuit. When the electron reaches the cathode, it combines with the H^+^ ion to generate H_2_ gas.

3 $$4{\mathrm{H}}^{+}+4{\mathrm{e}}^{-}\to 2{\mathrm{H}}_{2}$$

Conversely, the OH^-^ ion changes into H_2_O and releases O_2_ gas after it loses an electron and becomes unstable.

4 $$4 {\text{OH }} \to 2{\mathrm{H}}_{2} {\text{O }} + {\text{ O}}_{2}$$

The energy required to produce one kilogram of hydrogen (H_2_) from the water electrolysis process depends on many factors, such as the process efficiency and voltage used. Still, the average amount ranges between 50 and 55 kW of electrical energy.

B. Fuel cell

**Fig. 4:**
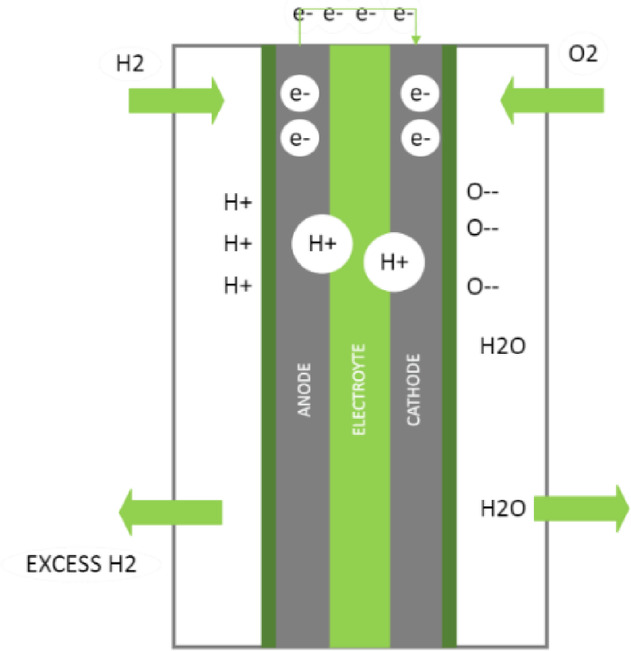
Fuel cell.

Despite the wide variety of fuel cell types, they all work with the same theory of operation. As shown in Fig. 4, there is a similarity between the fuel cell and the electolyser components as the fuel cell consists of two electrodes as well, the anode and the cathode, and the anode is called the hydrogen or fuel electrode, and the cathode is called the air or oxygen electrode. An external electrical circuit through which electrons travel connects the anode and the cathode. The electrodes are immersed in an electrolyte, as in the electrolyser, where ions are transferred. Among the important components of the fuel cell is the catalyst, as its presence is necessary to start the chemical reactions in the cell, and it may be a specific substance or just high temperature, depending on the type of fuel cell, which is often named according to the catalyst used in it.

When hydrogen is delivered into the fuel cell and reaches the catalyst-anode interface, it breaks into hydrogen ions and releases some electrons.

5 $${2\mathrm{H}}_{2}\to 4{\mathrm{H}}^{+}+4{e}^{-}$$

Next, as the electrons pass via an external electrical circuit, the hydrogen ions pass through the electrolyte. This is due to the electrolyte’s much greater impedance to electron flow than an external circuit. The electrons generate negative oxygen ions at the cathode when they are in the presence of oxygen.

6 $${\mathrm{O}}_{2}+4{e}^{-}\to {2O}^{-}$$

H_2_O is formed when these negative oxygen ions mix with the positive hydrogen ions that have moved through the electrolyte to the cathode.

7 $$4{\mathrm{H}}^{+}+{2O}^{-}\to 2{\mathrm{H}}_{2}\mathrm{O}$$

The amount of electrical energy generated from one kilogram of hydrogen (H_2_) varies according to the type of fuel cell, its efficiency and the purity of the hydrogen used, and if air or oxygen is used with it, and other factors. Still, it is estimated at an approximate value of about 33 kWh.

C. SOFC

**Fig. 5:**
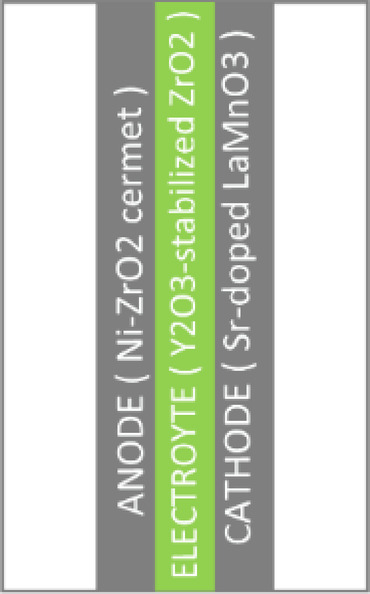
Solid oxide fuel cell materials.

In particular, the SOFC is described. The various components of this type of fuel cell are made of specific materials, as shown in Fig. 5:

- *Cathode (oxygen electrode)* Strontium-doped lanthanum manganate.
- *Electrolyte* Yttrium stabilized zirconia (YSZ).
- *Anode (fuel electrode)* Nickel metal, which is NiO in a composite with YSZ.
- *Catalyst* High operating temperature (600–1000 °C).

**Fig. 6:**
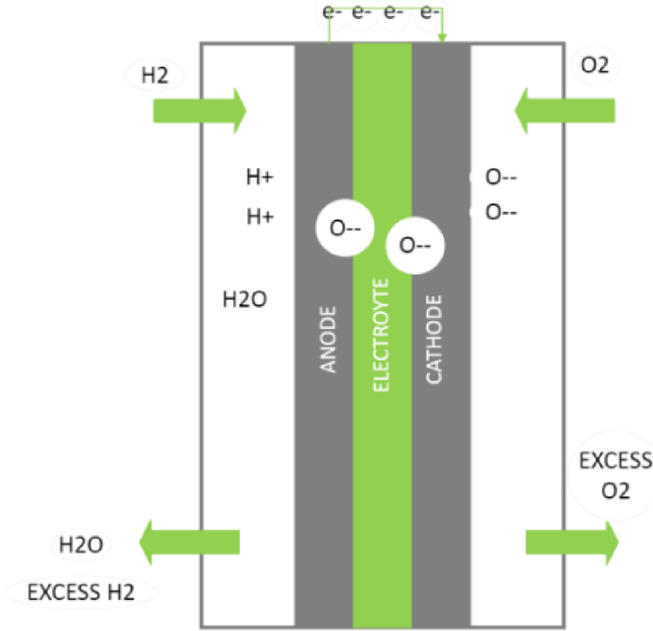
Solid oxide fuel cell operation.

The type of ions that pass through the electrolyte is a characteristic that makes the SOFC function differently. In SOFCs, the electrolyte is traversed by negative oxygen ions (oxide ions), as opposed to other fuel cells where the electrolyte is traversed by positive hydrogen ions (protons). This difference can be shown in Fig. 6.

### V-I Polarization curve

A set of mathematical equations shown below controls the relationship between the SOFC electric current and electrical potential and can be used to derive the V-I polarization curve^12^.

- *The Nernst voltage* The open circuit voltage without considering any losses in the cell.
- *Ohmic resistance* The voltage drop caused by electrons passing through the different cell components.
- *Activation polarization* The voltage loss to overcome the energy barrier of the electrochemical processes occurring within the cell.
- *Concentration polarization* The voltage loss due to variations between the partial pressure of the gas at the reaction site and the steam-hydrogen combination during gas diffusion while the cell operates at relatively higher current density values.

The Gibbs Free Energy of the water forming or splitting determines the Nernst Voltage.

8 $${E}^{o}= \frac{-\Delta \overline{{g }_{f}}^\circ }{2 F}$$

where ${E}^{o}$ - The standard cell potential when the concentrations of the reactants and products are at their standards (1 atm); $\Delta \overline{{g }_{f}}^\circ$ - The Gibbs Free Energy of water forming process, which occurs in the SOFC; $F$ - Faraday constant which equals 96,486 C/mol.

Additionally, the term “$\frac{RT}{2F}\mathrm{ln}\left(Q\right)$” is added to reflect the fact that the SOFC does not work under standard conditions. It is derived from statistical mechanics and characterizes how the system’s deviation from standard conditions contributes to the change in Gibbs free energy.

9 $$E= {E}^{o} - \frac{RT}{2F}\mathrm{ln}\left(Q\right)$$

where $E$ - The cell potential, also known as the Nernst voltage; $R$ - The universal gas constant that relates energy and temperature in the ideal gas law and equals 8.314 kJ/kmol; $T$ - The absolute temperature in the reaction site in Kelvin; Q - The reaction quotient which is a ratio between the amounts of chemical reaction products and the amounts of chemical reaction reactants.

10 $$E= {E}^{o} - \frac{RT}{2F}\mathrm{ln}\left(\frac{{[{\mathrm{H}}_{2}\mathrm{O}]}^{1} }{{[{\mathrm{H}}_{2}]}^{1} *{[{\text{ O}}_{2}]}^\frac{1}{2}}\right)$$

It depends on the reaction:

11 $${\mathrm{H}}_{2}+{ \frac{1}{2}\text{ O}}_{2}\to {\mathrm{H}}_{2}\mathrm{O}$$

As that [ ] means the molar concentrations of the products/reactants and the power depends on the balance of the chemical equation.

The ohmic voltage losses value depends on Ohm’s law.

12 $${V}_{ohmic}=r* {I}_{fc}$$

where $\mathrm{r}$ - Resistance to the ions flowing in the electrolyte and the resistance to the electrons flowing through the electrode materials

The Butler-Volmer equation for electrochemical kinetics has been used to derive the Tafel equation. And the Tafel equation is the equation for calculating the activation voltage loss value in the SOFC.

The Butler-Volmer equation is as follows:

13 $${I}_{fc}={I}_{0}\left\{exp\left(\beta \frac{nF{V}_{act}}{RT}\right)-exp\left[-(1-\beta )\frac{nF{V}_{\text{act }}}{RT}\right]\right\}$$

where ${I}_{fc}$ - The fuel cell current density; ${I}_{0}$ - The exchange current density, and its value depends on the nature of the reactants, the electrode material, and the temperature; β - The charge transfer coefficient; ${V}_{act}$ - The activation overpotential; n - The number of moles of electrons transferred.

The Tafel equation is as follows:

14 $${V}_{\text{act }}= \frac{- RT}{F}\mathrm{ln}\left({I}_{0}\right)+ \frac{RT}{F} \mathrm{ln}\left({I}_{fc}\right)$$

The Concentration Voltage Loss is derived from the Nernst equation.

15 $${V}_{con}=- \frac{RT}{2F}\mathrm{ln}\left(1- \frac{{I}_{fc}}{{I}_{L}}\right)$$

where ${I}_{L}$ - The limiting current density, which is the maximum fuel cell current density that can be maintained without concentration losses.

Concentration losses may be significant at relatively high current densities due to the possibility of reactant gas consumption exceeding supply.

So, the SOFC voltage can be calculated by

16 $${V}_{fc}= E- {V}_{ohmic}- {V}_{\text{act }}- {V}_{con}$$

### Dynamic model

The dynamic model that shows how the voltage of a cell changes when the electric current drawn from it changes depends on a set of principles that can be reviewed as follows:^23^

1. Electrochemical model:

This model uses a balance equation that describes the change that occurs in the concentration of each chemical reaction component. This balance equation can be expressed using the flow rate of the input, the flow rate of the output, and the exit molarity.

17 $$\frac{PV}{RT}\frac{d}{dt}{x}_{i}={N}_{i}^{\mathrm{in}}-{N}_{i}^{o}+{N}_{i}^{r}$$

where $$V$$ - The cell volume $$\left({\mathrm{m}}^{3}\right)$$; $${N}_{i}^{\text{in }},{N}_{i}^{o}$$ - Molar flow rates (mole/s) of the $$i$$ ^th^ reactant at the cell input and output, respectively; $${N}_{i}^{r}$$ - The reaction rate $$($$ mole/s) of the $$i$$ th reactant; $$T$$ - The cell temperature in $$\mathrm{K}; P$$ - The cell pressure $$($$atm$$); R$$ - The gas constant $$\left(8.31 \, \mathrm{J}/\mathrm{mole}.\mathrm{K}\right)$$.

Using the previous equation and applying it to the reaction shown in Eq. (11) and then using the Laplace transform, the following three equations of ${\mathrm{H}}_{2}$, ${\mathrm{O}}_{2}$ and ${\mathrm{H}}_{2}O$ can be deduced and can be used directly in the model.

18 $$\begin{array}{cc}{x}_{\mathrm{H}2}\left(s\right)& =\frac{\frac{1}{{K}_{\mathrm{H}2}}}{1+{\tau }_{\mathrm{H}2}*s}\left({N}_{\mathrm{H}2}^{\mathrm{in}}-2{K}_{r}I\right) ,where {\tau }_{\mathrm{H}2}= \frac{V}{{K}_{\mathrm{H}2}RT}\\ & \end{array}$$

19 $$\begin{array}{cc}{x}_{\mathrm{O}2}\left(s\right)& =\frac{\frac{1}{{K}_{\mathrm{O}2}}}{1+{\tau }_{\mathrm{O}2}*s}\left({N}_{\mathrm{O}2}^{\mathrm{in}}-2{K}_{r}I\right) ,where {\tau }_{\mathrm{O}2}= \frac{V}{{K}_{\mathrm{O}2}RT}\\ & \end{array}$$

20 $$\begin{array}{cc}{x}_{\mathrm{H}2\mathrm{O}}\left(s\right)& =\frac{\frac{1}{{K}_{\mathrm{H}2\mathrm{O}}}}{1+{\tau }_{\mathrm{H}2\mathrm{O}}*s}\left({N}_{\mathrm{H}2\mathrm{O}}^{\mathrm{in}}-2{K}_{r}I\right) ,where {\tau }_{\mathrm{H}2\mathrm{O}}= \frac{V}{{K}_{\mathrm{H}2\mathrm{O}}RT}\\ & \end{array}$$

where ${N}_{\mathrm{H}2}^{\mathrm{in}}$ - Input hydrogen flow $(\mathrm{kmol}/\mathrm{s})$; ${K}_{\mathrm{H}2}$ - Hydrogen valve molar constants; ${x}_{\mathrm{H}2}$ - Mole fractions of hydrogen; ${\tau }_{\mathrm{H}2}$ - The time constant associated with the hydrogen flow (sec)$; {K}_{r}$ - A constant depending on Faraday’s constant and number of electrons (N) in the reaction where ${K}_{r}=\frac{N}{4F}$.

2. Temperature model:

Since the temperature has a direct and noticeable effect on the amount of electrical power generated from the fuel cell, an energy balance equation can be used as follows:

21 $${M}_{p}{C}_{P}\frac{dT}{dt}={q}_{e}{V}_{e}+\sum {Q}_{i}$$

where ${M}_{p}$ - Mass of the cell unit (in kilograms); ${V}_{e}$ - Volume of the cell unit (${\mathrm{m}}^{3}$); ${C}_{P}$ - Heat capacity of the cell unit (in Joules per kilogram. Kelvin); $T$ - Cell temperature in $\mathrm{K}; {q}_{e}$ - Heat generated from the electrochemical reaction per volume unit; ${Q}_{i}$ - Total heat $\left(J\right).$

Using the previous equation, the following equation can be deduced and can be used directly in the model.

22 $${T}_{out}= {T}_{in}+ \frac{V*I*Thickness*\frac{(1-\eta )}{\eta }}{\frac{\text{Thermal Conductivity }*\text{ dt}}{\alpha * \mathrm{density}* {Thickness}^{2}*(1+n*{T}_{in}+b* {{T}_{in}}^{2})}}$$

3. Voltage activation, concentration, and ohmic losses: Nernst voltage equation.

Depending on all the previous equations mentioned in this section, the model of the SOFC can be built.

## Integrated modeling–analysis–optimization methodology

This section presents the integrated modeling and analysis methodology adopted in this study. It includes the formulation of the SOFC model, the derivation of steady-state and dynamic performance characteristics, and the application of optimization techniques for model calibration and operating-condition-dependent analysis. Specifically, this section covers the following aspects:

- Development of the SOFC model based on the relevant electrochemical and operating parameters.
- Formulation and analysis of the voltage–current (V–I) polarization characteristics.
- Investigation of the impact of operating conditions, such as temperature and reactant flow rates, on steady-state performance.
- Analysis of the dynamic voltage response under step changes in electrical current.
- Optimization-based parameter tuning to align the model predictions with published experimental benchmark data.
- Assessment of the influence of operating conditions on dynamic response and generated power output.

A. The voltage–current polarization curves

In this part, two models of two different types of SOFC will be built using MATLAB based on the equations reviewed in the previous section, thus deducing the V-I polarization curve for each type.

### Conventional SOFC

The first SOFC to be studied in this part is a conventional SOFC used in electricity generation only. It is of the tubular type and anode-supported and built by NanoDynamics Inc. in New York. In Table 1, its parameters are stated^12^.

**Table 1:** Parameters of the SOFC under study.

Parameters	Values	Units
Hydrogen flow rate (N_H2_ )	31, 41, 51	mL/min
Temperature (T)	(750, 800, 850) + 273	K
${E}^{o}$	1.1	volt
R	0.0257	Ω
${K}_{L}$	69	–
$A$	101.2	kA/cm2
${E}_{\mathrm{act}}$	120	kJ/mol

There are several parameters that affect the value of ${I}_{L}$, such as material features, temperature, and reactant concentration. It can be calculated as follows:

23 $${I}_{L}={K}_{L}\frac{\mathrm{ln}\left(1-{p}_{\mathrm{re}}\right)}{T}$$

where ${K}_{L}$ - A constant factor; ${p}_{\mathrm{re}}$ - The reactant concentration.

The value of ${I}_{0}$ can be calculated by:

24 $${I}_{0}=A{e}^{-{E}_{\text{act }}/RT}$$

where $\mathrm{A}$ - A pre-exponential factor; ${E}_{\mathrm{act}}$ - The activation energy of the electrochemical reaction.

The V-I polarization curves at various temperatures and flow rates are displayed in the following figures.

**Fig. 7:**
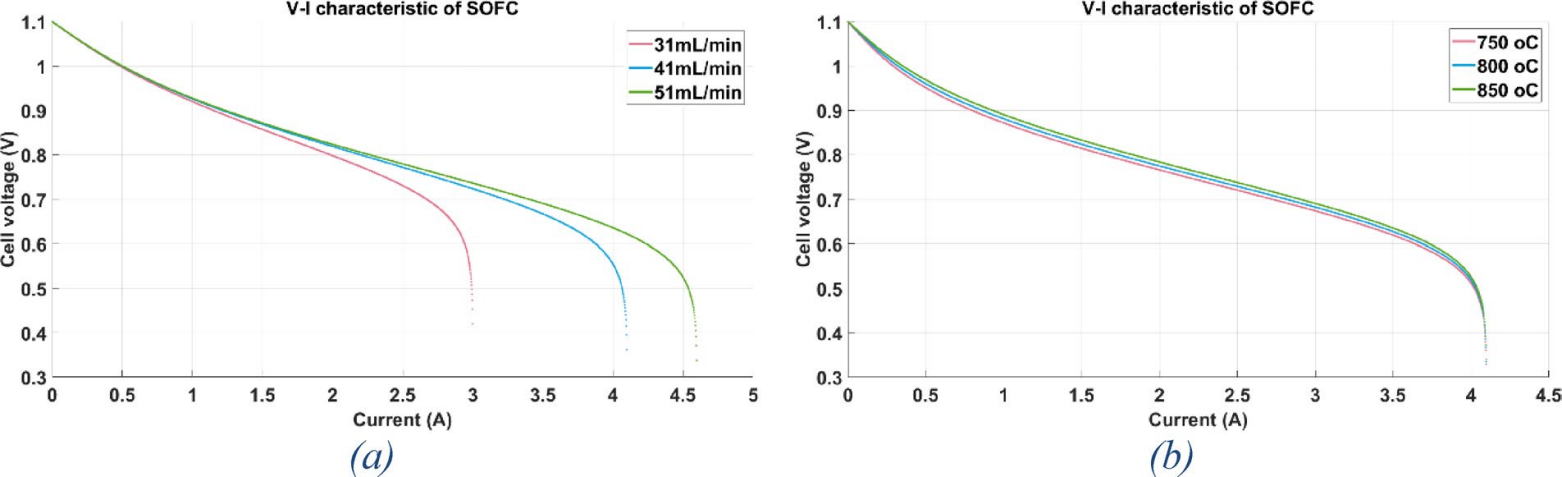
(**a**) V-I polarization curve at H2 flow rate = 31, 41, 51 mL/min and 850 °C. (**b**) V-I polarization curve at H2 flow rate = 41 mL/min and 750, 800, 850 °C.

Comparing various hydrogen flow rates at the same temperature (850 °C) is shown in Fig. 7a.

A comparison of various operating temperatures at the same hydrogen flow rate (41 mL/min) is shown in Fig. 7b.

Based on the above figure, it is deduced that at a certain flow rate, the cell voltage reduces when the fuel cell temperature drops, and vice versa. A decrease in flow rate for the same temperature causes a corresponding decrease in cell voltage and vice versa.

### Reversible SOFC

The second SOFC to be studied in this part is a rSOFC used in Hydrogen production when operated in electroyser mode and electricity generation when operated in fuel cell mode. It is built within the scope of the HyBCN project at the IREC’s facilities. In Table 2, its parameters are stated^38^.

**Table 2:** Parameters of the reversible SOFC under study.

Parameters	Values	Units
Area	50	${\mathrm{cm}}^{2}$
$T$	Variable	$K$
${E}_{\mathrm{act}}$	120	$\mathrm{kJ}/\mathrm{mol}$
${\gamma }_{C}$	415,500	$\mathrm{A}/{\mathrm{cm}}^{2}$
${\gamma }_{A}$	600,000	$\mathrm{A}/{\mathrm{cm}}^{2}$
${\alpha }_{c}$ and ${\alpha }_{A}$	0.3 and 0.5	–
$r$	0.165	$\Omega /{\mathrm{cm}}^{2}$
${D}_{{O}_{2}}^{\mathrm{eff}}$, ${D}_{{H}_{2}}^{\mathrm{eff}}$ and ${D}_{{H}_{2}\text{O }}^{\mathrm{eff}}$	0.0228, 0.0927 and 0.0436	${\mathrm{cm}}^{2}/\mathrm{s}$
${L}_{F}$	0.04	${\mathrm{cm}}^{2}$
${L}_{A}$	0.007	$\mathrm{cm}$

**Fig. 8:**
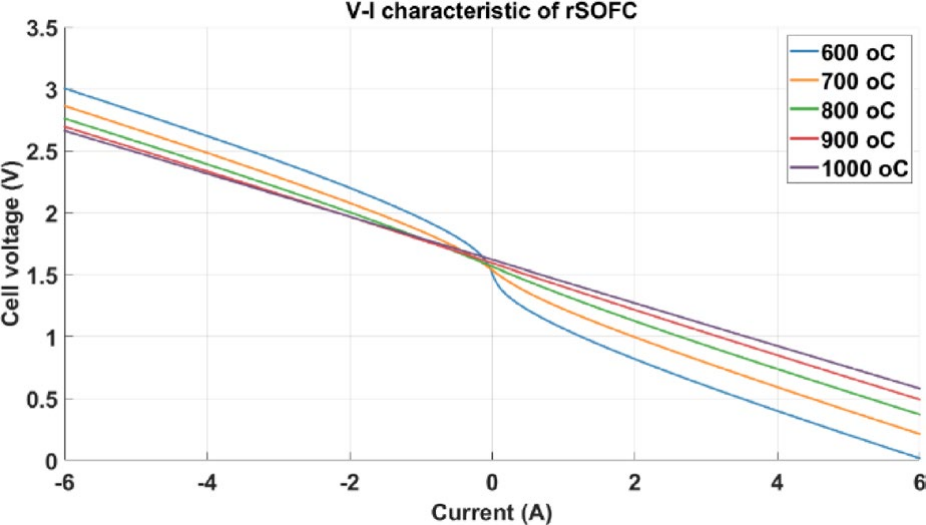
V-I polarization curve at H2 flow rate = 18.16 L/min and 600, 700, 800, 900, 1000 °C*.*

Its V-I polarization curves are shown in Fig. 8 at various temperatures: 600, 700, 800, 900, and 1000 °C with the same hydrogen flow rate (18.16 L/min).

Based on the figure, it can be observed that for a certain flow rate, a reduction in cell temperature results in a corresponding decrease in cell voltage. This is the conclusion for the fuel cell mode. In contrast, increasing the temperature lowers the voltage needed in the electrolyser mode.

B. The dynamic response

In this part, a dynamic model of two different types of SOFC is discussed depending on the equations discussed in the previous section to be applied to studies on dynamic stability. The change in the output voltage of the cell, which follows the change in the drawn current from it, is shown.

### Conventional SOFC

The first SOFC to be studied in dynamic modelling is a conventional 100 kW standalone SOFC stack. In Table 3, the steady-state data of the stack under study is stated^14^^23^.

**Table 3:** The steady-state data for a stand-alone 100-kW SOFC stack.

Parameters	Values	Units
Rated power	$100$	$\mathrm{kW}$
Rated stack voltage	$286.3$	$\mathrm{V}$
Rated stack current	$300$	$\mathrm{A}$
Number of cells	384	–
Number of stacks	1	–
Open circuit voltage for each cell	$0.935$	$\mathrm{V}$
Input fuel flow (Fuel)	$1.2*{10}^{-3}$	$\mathrm{kmol}/\mathrm{s}$
Input air flow (Air)	$2.4*{10}^{-3}$	$\mathrm{kmol}/\mathrm{s}$
Tafel slope(b)	0.11	–
Tafel constant (a)	0.05	–
Cell area	$1000$	${\mathrm{cm}}^{2}$
Operating point cell temperature $\left( {\mathrm{T}} \right)$	$1000$	$^\circ {\mathrm{C}}$
Ohmic resistance constant $\left( \beta \right)$	− 2870	–
Ohmic resistance constant $\left( \alpha \right)$	0.2	–
Constant temperature $\left( {T_{0} T0} \right)$	$923$	$^\circ {\mathrm{C}}$
Thickness ($h_{eff}$)	$5$	${\mathrm{mm}}$
Thermal conductivity ($I_{s}$)	$15$	${\mathrm{W}}/\left( {{\mathrm{mK}}} \right)$
Efficiency (eta)	$80$	${{\% }}$
Density (ro)	$7000$	${\mathrm{kg}}/{\mathrm{m}}^{3}$
Limiting current ($I_{L}$)	$0.8$	$\mathrm{A}/{\mathrm{m}}^{2}$
${K}_{\mathrm{H}2}$	$8.4*{10}^{-4}$	$\mathrm{kmol}/(\mathrm{atms})$
${K}_{\mathrm{O}2}$	$2.5*{10}^{-3}$	$\mathrm{kmol}/(\mathrm{atms})$
${K}_{\mathrm{H}2\mathrm{O}}$	$2.8*{10}^{-4}$	$\mathrm{kmol}/(\mathrm{atms})$
${\tau }_{\mathrm{H}2}$	26.1	$\mathrm{s}$
${\tau }_{\mathrm{O}2}$	2.91	$\mathrm{s}$
${\tau }_{\mathrm{H}2\mathrm{O}}$	78.3	$\mathrm{s}$

**Fig. 9:**
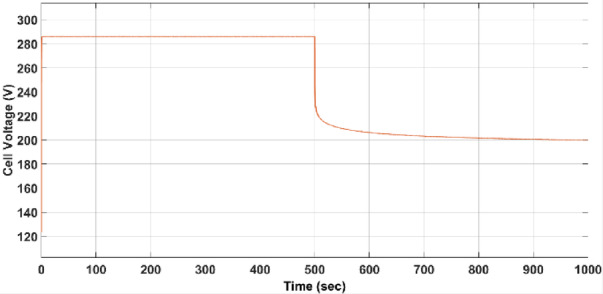
Dynamic response of 100-kW SOFC stack.

The fuel cell stack’s output voltage is shown in Fig. 9, following a step change in the current drawn from the stack from 300 A (rated value) to 500 A.

As shown in Fig. 9, the voltage decreases from 286 V (rated value) to 200 V within approximately 460 s.

### Reversible SOFC

**Fig. 10:**
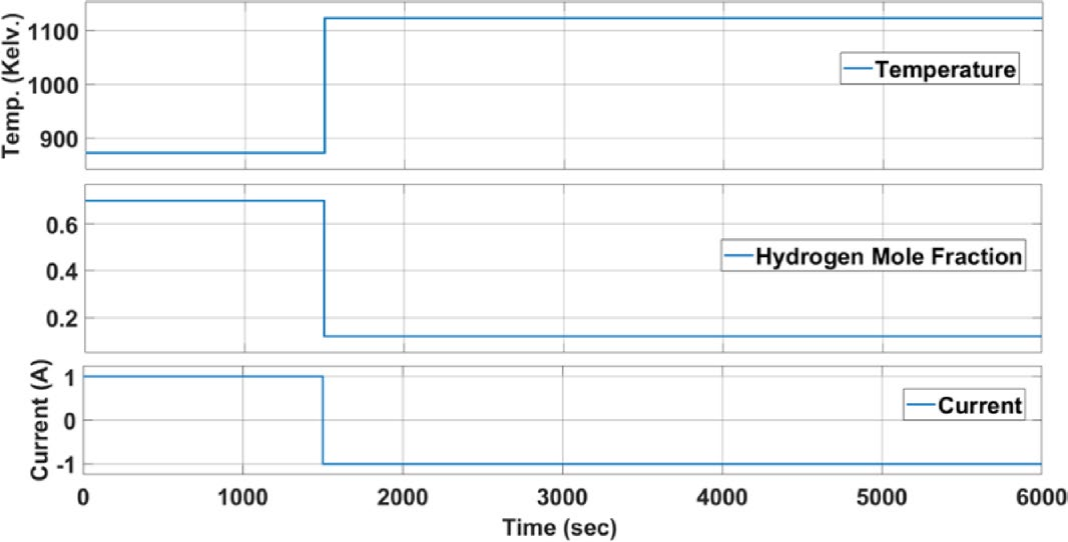
boundary conditions characterizing the steady-state operation points for both the fuel cell and electrolyser modes.

The second SOFC to be studied in this part is the same rSOFC studied in the V-I polarization curve part^38^. In this study, the operating mode of rSOFC will be switched from the fuel cell mode to the electrolyser mode and assess how the cell behaves in this case. The operating conditions in the two modes are shown in Table 4 and Fig. 10.

**Table 4:** boundary conditions characterizing the steady-state operation points for both the fuel cell and electrolyser modes.

	Before switching	After switching
Mode of operation	fuel cell mode	electroyser mode
Hydrogen mole fraction / partial pressure	70%	12%
Temperature	600 °C	850 °C
Current	1A	− 1A

Since the chemical reaction that occurs in the case of electroyser mode is an endothermic reaction, the operating temperature will be raised when switching occurs. By adjusting their flow rate, the reactant composition is changed step-wise from 70 mol fraction hydrogen and 30 mol fraction water to 12 mol fraction hydrogen and 88 mol fraction water. Hydrogen and vapor will be mixed before entering the cell to prevent oxidation of the nickel in the fuel electrode.

**Fig. 11:**
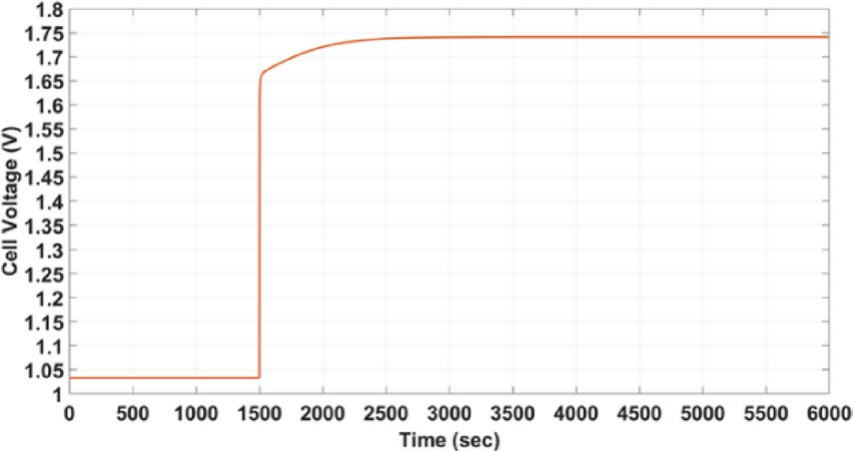
dynamic response of rSOFC.

The rSOFC’s output voltage is shown in Fig. 11 during the mode transition.

As seen in Fig. 11, it is observed that the voltage value changes from 1.0329 to 1.7411 V in 1844s (31 min).

C. Modelling optimization

In this section, certain parameters will be re-calculated, and the operational conditions will be adjusted to optimize the modeling of the fuel cell.

### Optimization-based model calibration using experimental data

When comparing the lab results of the conventional SOFC published in reference^12^ with the results of the modelling in the same reference and the results of the modelling in this study, it is found that the relative error is 0.0099 and 0.011437, respectively, but this error can be reduced by modifying the values of the parameters used in the modelling mentioned in Table 1, which makes the model more consistent with the lab results.

The experimental data used for model validation are extracted from the study reported in^12^, which provides detailed laboratory measurements of a conventional SOFC under multiple operating conditions. The validation in the present work is based on a direct comparison with the experimental polarization curves reported in Figs. 7, 8, and 10 of^12^, with particular emphasis on the reference operating condition of 800 °C and a reactant flow rate of 41 ml/min. This allows the relative modeling error to be quantitatively assessed across a representative operating range. This approach ensures a reliable and literature-consistent validation of the proposed model without restricting the comparison to a single operating point.

In Table 1, there are a number of parameters, but not all of them will be considered in this study. Only the standard cell potential (${E}^{o}$), the constant factor in the limiting current density equation (${K}_{L}$), and the pre-exponential factor in the exchange current density equation ($A$) will be studied since the value of R is determined by the characteristics of the materials used in fabricating the cell, and the value of ${E}_{\text{act }}$ is determined by the chemical reactions that take place inside the cell.

#### The standard cell potential (${{\boldsymbol{E}}}^{{\boldsymbol{o}}}$)

In the model applied in this study, a constant value of ${E}^{o}$ is used^12^. It is 1.1 V, but Eq. (8) shows that ${E}^{o}$ depends on the value of Gibbs Free Energy for the process of water formation, and through what has been studied previously Gibbs Free Energy is affected by temperature change, so an empirical relationship was concluded linking ${E}^{o}$ and the temperature, which is^39^:

25 $${E}^{o}= -0.0002809002*T+1.2770578798$$

When using this equation instead of using the constant value of 1.1 V, it is found that the relative error value increased to 0.1611, which means that using the constant value gives a better and more accurate model.

#### The constant factor in the limiting current density equation (${K}_{L}$)

${K}_{L}$ Is a constant factor used in the calculation of concentration voltage loss, whose effect appears significantly at the high current density values of the cell, as previously explained through equations and model results. When trying to change the ${K}_{L}$ value, the results of the model become significantly different from those of the lab. When its value increases, the cell model gives much higher current values than what can actually be drawn from the cell and vice versa. As a result, the value of ${K}_{L}$ will not be changed.

#### The pre-exponential factor in the exchange current density equation ($A$)

$A$ Is the pre-exponential factor used in the calculation of activation voltage loss. The value of $A$ mentioned in Table 1 is determined through curve fitting. To minimize the relative error, this value can be adjusted. Four different optimization techniques are employed, and their results are compared to achieve the lowest possible relative error.

This study does not aim to introduce a new metaheuristic optimization algorithm. Instead, it investigates the use of established optimization techniques within a unified framework for SOFC model calibration and operating-condition-dependent performance analysis.

*Optimization techniques*:

- Teaching–Learning Based Optimization (TLBO) Technique^40^

**Fig. 12:**
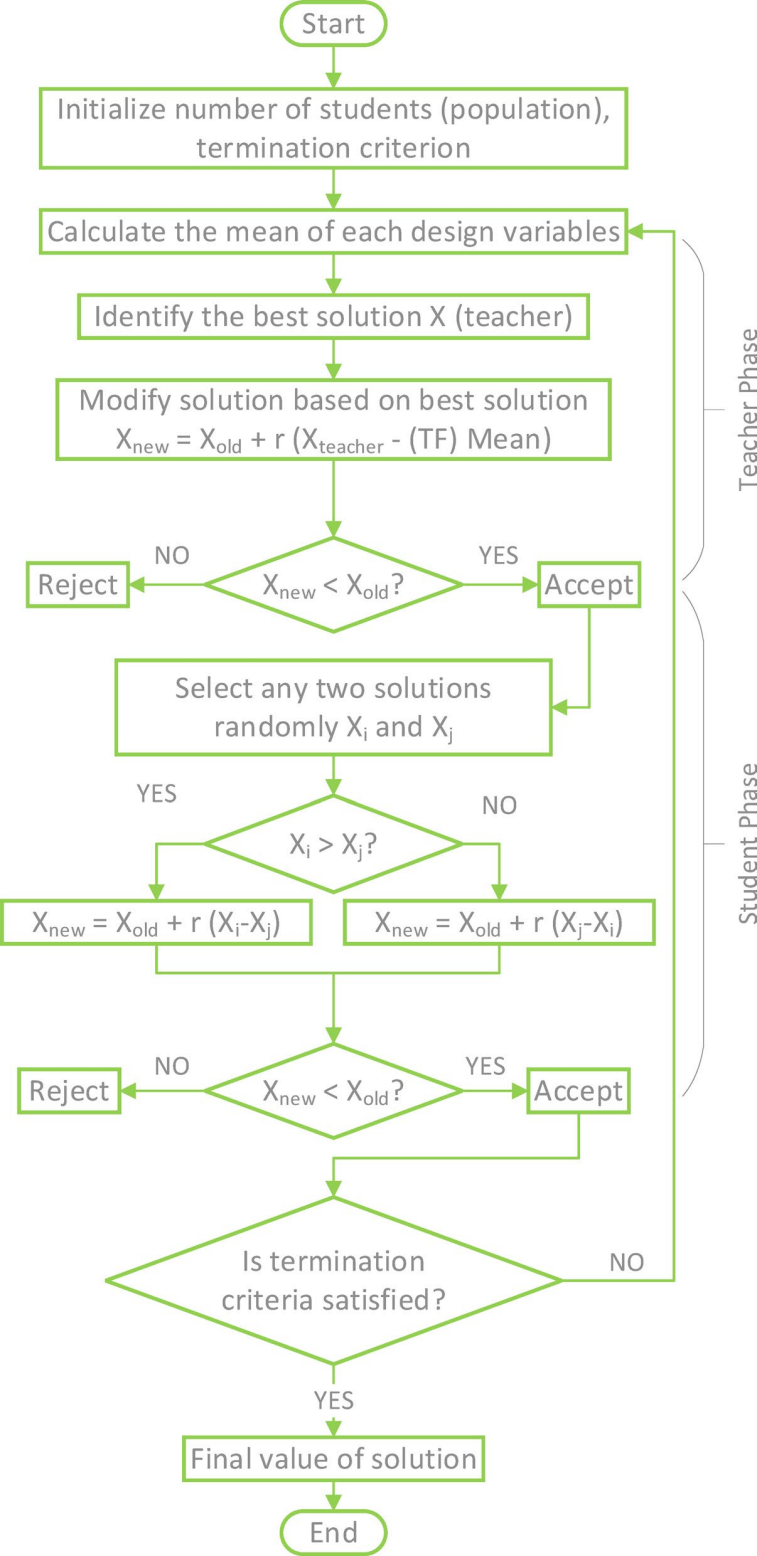
Flowchart of TLBO.

The TLBO technique is one of the most famous, accurate, and widespread AI-based optimization techniques that simulate natural processes, and this technique simulates the learning process. This technique depends on two basic stages, namely the teacher stage and the student stage. At the teacher stage, the teacher is the main source of learning in the class, and at the student stage, communication between students and the exchange of ideas between them is the source of learning. The MATLAB code for this technique has been published in 2015. S. Heris developed it as part of the work of the Yarpiz team. The equations and parameters on which the code is built are shown in the flowchart in Fig. 12.

- Chaos Game Optimization (CGO)^41,42^

CGO is inspired by chaos theory, which is a distinctive way to search for the best solution to complex problems, and this chaos theory can be seen in nature around us, such as the movement of butterflies that appear as random, but they go according to a certain system and can lead to major changes, and this is what happens in CGO, as the initial solutions greatly affect the final solution.

CGO works by choosing several random solutions in the search area, then it is started through these initial solutions to reach new solutions that seem to be random, but it goes according to a specific system, where a triangle is determined around each of the initial solutions, and one of the vertices of the triangle is chosen, so the initial solution moves half the distance between it and this vertex and a new point or solution arises, then the new solution begins to do The same step as the previous one by moving half the distance to one of the vertices of the triangle and creating a new solution, and so on. This triangle is called the Sierpinski triangle, which is one of the famous fractals that repeat themselves in the same pattern on any scale. To reach the final solution, which represents the best solution to the problem, the solutions are compared, and the worst is excluded until the difference is negligible or the number of specific iterations expires.

**Fig. 13:**
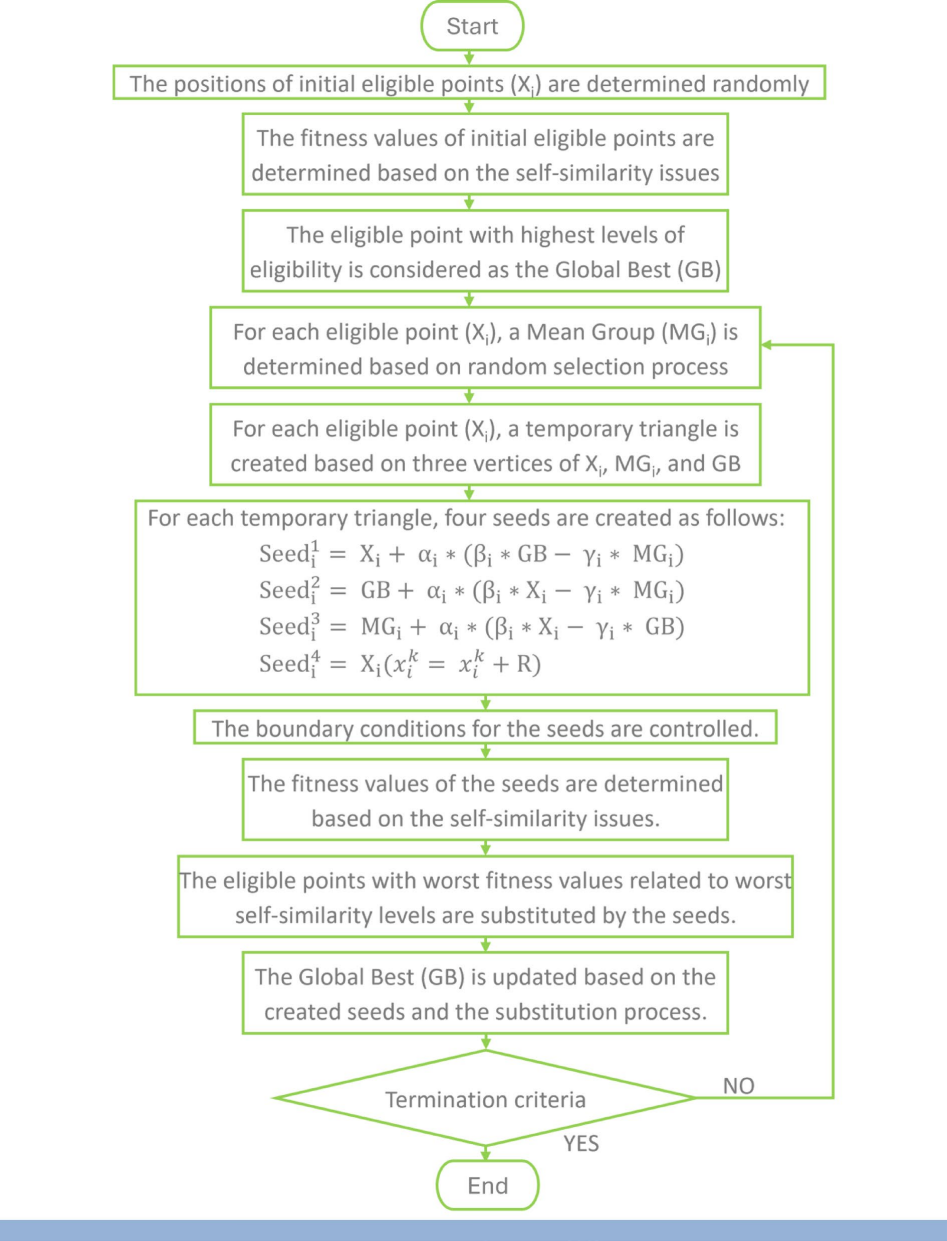
Flowchart of CGO.

In 2020, the CGO MATLAB code has been published by Siamak Talatahari. The equations and parameters on which the code is built are shown in the flowchart in Fig. 13.

- Secretary Bird Optimization Algorithm (SBOA)^43,44^

**Fig. 14:**
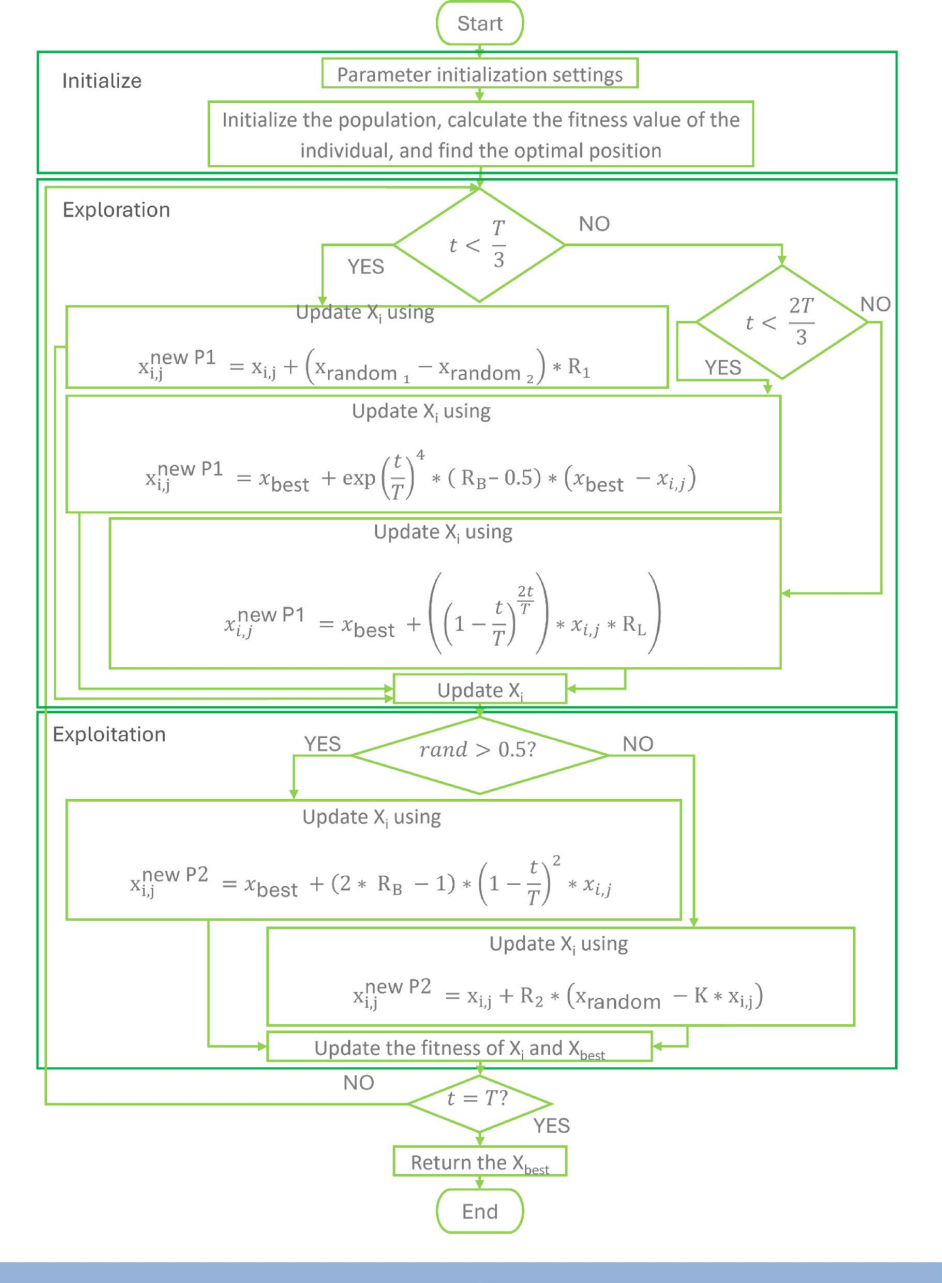
Flowchart of SBOA.

SBOA is a metaheuristic optimization technique inspired by the behavior of Secretary Bird during hunting and escaping from predators. This bird is well-known for its strength, speed, and intelligence during hunting. This algorithm can be divided into three stages: Initial Preparation, Exploration Phase, and Exploitation Phase. In Initial Preparation, several random solutions are generated, each of which represents a set of variables to be optimized and can be evaluated using the objective function. In Exploration Phase, the bird explores the prey by changing its position randomly until reaching the best solution. In Exploitation Phase, the bird escapes from the predators using two techniques: Camouflage and Flight/Run. The MATLAB code has been published in April 2024. The flowchart describing the technique is shown in Fig. 14.

- Walrus Optimization Algorithm (WaOA)^45,46^

WaOA is one of the algorithms that simulate something in nature around us, which is the behavior of the walrus. It is one of the social animals that live in a herd in polar places and have a leader, and as usual in nature, it is the strongest in the herd. The WaOA depends on three basic behaviors of the walrus: the search for food led by the strongest in the herd, migration to new places, and escape from predators. These three behaviors make WaOA one of the optimization algorithms that strikes a balance between exploitation and exploration.

*Mathematical modelling of WaOA:*

Each walrus represents a possible solution to the optimization problem and is used as a searcher for the optimal solution among all existing solutions in the search area, where it is possible through the walrus position in the search area to determine the possible values of the variables that constitute the problem to be solved. Accordingly, the population matrix can be represented by Eq. (26), knowing that walrus populations are randomly selected at the beginning of the implementation of WaOA.

26 $$X={\left[\begin{array}{c}{X}_{1}\\ \vdots \\ {X}_{i}\\ \vdots \\ {X}_{N}\end{array}\right]}_{N\times m}={\left[\begin{array}{lllll}{x}_{\mathrm{1,1}}& \cdots & {x}_{1,j}& \cdots & {x}_{1,m}\\ \vdots & \ddots & \vdots & .& \vdots \\ {x}_{i,1}& \cdots & {x}_{i,j}& \cdots & {x}_{i,m}\\ \vdots & \ddots & \vdots & .& \vdots \\ {x}_{N,1}& \cdots & {x}_{N,j}& \cdots & {x}_{N,m}\end{array}\right]}_{N\times m}$$

where $X$ - The population of walruses; ${X}_{i}$ - The $i$ th walrus / candidate solution; ${x}_{i,j}$ - The value of the $j$ th decision variable suggested by the $i$ th walrus; $N$ - The number of walruses / candidate solutions; $m$ - The number of decision variables.

The objective function is then calculated based on the resulting values from each walrus in calculating the variables of the problem to be solved and then evaluated. This is calculated according to Eq. (27).

27 $$F={\left[\begin{array}{c}{F}_{1}\\ \vdots \\ {F}_{i}\\ \vdots \\ {F}_{N}\end{array}\right]}_{N\times 1}={\left[\begin{array}{c}F\left({X}_{1}\right)\\ \vdots \\ F\left({X}_{i}\right)\\ \vdots \\ F\left({X}_{N}\right)\end{array}\right]}_{N\times 1}$$

where $F$ - The objective function vector; ${F}_{i}$ - The value of the objective function evaluated based on the $i$ th walrus.

The best and worst walrus are determined based on the resulting values of the objective function calculated in Eq. (27), and in each iteration, the values of the objective function are updated, and thus, the best and worst walrus are updated as well.

Three phases represent the process of updating the walruses’ position in WaOA based on the three natural behaviors of the Walrus:

1. Phase 1, Feeding under leadership (exploration): A new position in the search area is determined for the walrus for the first time according to Eq. (28), and either the new position at this phase is kept or returned to the old position that was chosen randomly, and this is done based on the value of the objective function resulting from each of them and this comparison can be represented by Eq. (29).

28 $${x}_{i,j}^{{P}_{1}}={x}_{i,j}+{\mathrm{rand}}_{i,j}\cdot \left(S{W}_{j}-{I}_{i,j}\cdot {x}_{i,j}\right)$$

29 $${X}_{i}=\left\{\begin{array}{cc}{X}_{i}^{{P}_{1}},& {F}_{i}^{{P}_{1}}<{F}_{i},\\ {X}_{i},& \text{ else },\end{array}\right.$$

where ${X}_{i}^{{P}_{1}}$ - The new generated location for the $i$ th walrus based on the 1st phase; ${x}_{i,j}^{{P}_{1}}$ - Its $j$ th dimension; ${F}_{i}^{{P}_{1}}$ - Its objective function value; ${\mathrm{rand}}_{i,j}$ - Random numbers from the interval [0,1]; $SW$ - The best member; ${I}_{i,j}$ - It is a number to increase the exploration of the algorithm, usually 1 or 2

2. Phase 2, Migration: This phase depends on creating a new position for the walrus by moving to another walrus located in the search area based on Eq. (30). Also, the comparison between the two positions is the same as in phase 1, and the selection of the position that gives the largest value of the objective function and the exclusion of the other position is according to Eq. (31).

30 $${x}_{i,j}^{{P}_{2}}=\left\{\begin{array}{c}{x}_{i,j}+{\mathrm{rand}}_{i,j}\cdot \left({x}_{k,j}-{I}_{i,j}\cdot {x}_{i,j}\right),{F}_{k}<{F}_{i};\\ {x}_{i,j}+{\text{ rand }}_{i,j}\cdot \left({x}_{i,j}-{x}_{k,j}\right), \, {\mathrm{e}}{\mathrm{l}}{\mathrm{s}}{\mathrm{e}} \, ,\end{array}\right.$$

31 $${X}_{i}=\left\{\begin{array}{cc}{X}_{i}^{{P}_{2}},& {F}_{i}^{{P}_{2}}<{F}_{i};\\ {X}_{i},& \text{ else },\end{array}\right.$$

where ${X}_{i}^{{P}_{2}}$ - The new generated location for the $i$ th walrus based on the 2nd phase; ${x}_{i,j}^{{P}_{2}}$ - Its $j$ th dimension; ${F}_{i}^{{P}_{2}}$ - Its objective function value; ${X}_{k} - k \in \left\{ \mathrm{1,2},\dots \dots ,N\right\}$ and $k \ne i$. The selected walrus’s location to migrate the $i$ th walrus towards it; ${x}_{k,j}$ - Its $j$ th dimension; ${F}_{k}$ - Its objective function value.

3. Phase 3, Fighting or fleeing from predators (exploitation): For every existing walrus around which there is a neighborhood, a new position is created randomly within this neighborhood using Eqs. (32), and (33), and comparison between the two positions is based on Eq. (34) to choose the best of them based on the value of the objective function.

32 $${x}_{i,j}^{{P}_{3}}={x}_{i,j}+\left(l{b}_{local,j}^{t}+\left(u{b}_{local,j}^{t}-\text{ rand }\cdot l{b}_{local,j}^{t}\right)\right),$$

33 $$\text{Local bounds }:\left\{\begin{array}{c}l{b}_{local,j}^{t}=\frac{l{b}_{j}}{t},\\ u{b}_{local,j}^{t}=\frac{u{b}_{j}}{t},\end{array}\right.$$

34 $${X}_{i}=\left\{\begin{array}{cc}{X}_{i}^{{P}_{3}},& {F}_{i}^{{P}_{3}}<{F}_{i};\\ {X}_{i},& \text{ else },\end{array}\right.$$

where ${X}_{i}^{{P}_{3}}$ - The new generated location for the $i$ th walrus based on the 3rd phase; ${x}_{i,j}^{{P}_{3}}$ - Its $j$ th dimension; ${F}_{i}^{{P}_{3}}$ - Its objective function value; $t$ - Iteration counter; $l{b}_{j}$ and u ${b}_{j}$ - The lower and upper bounds of the $j$ th variable; $l{b}_{local,j}^{t}$ and $u{b}_{local,j}^{t}$ - local lower and local upper bounds allowable for the $j$ th variable

**Fig. 15:**
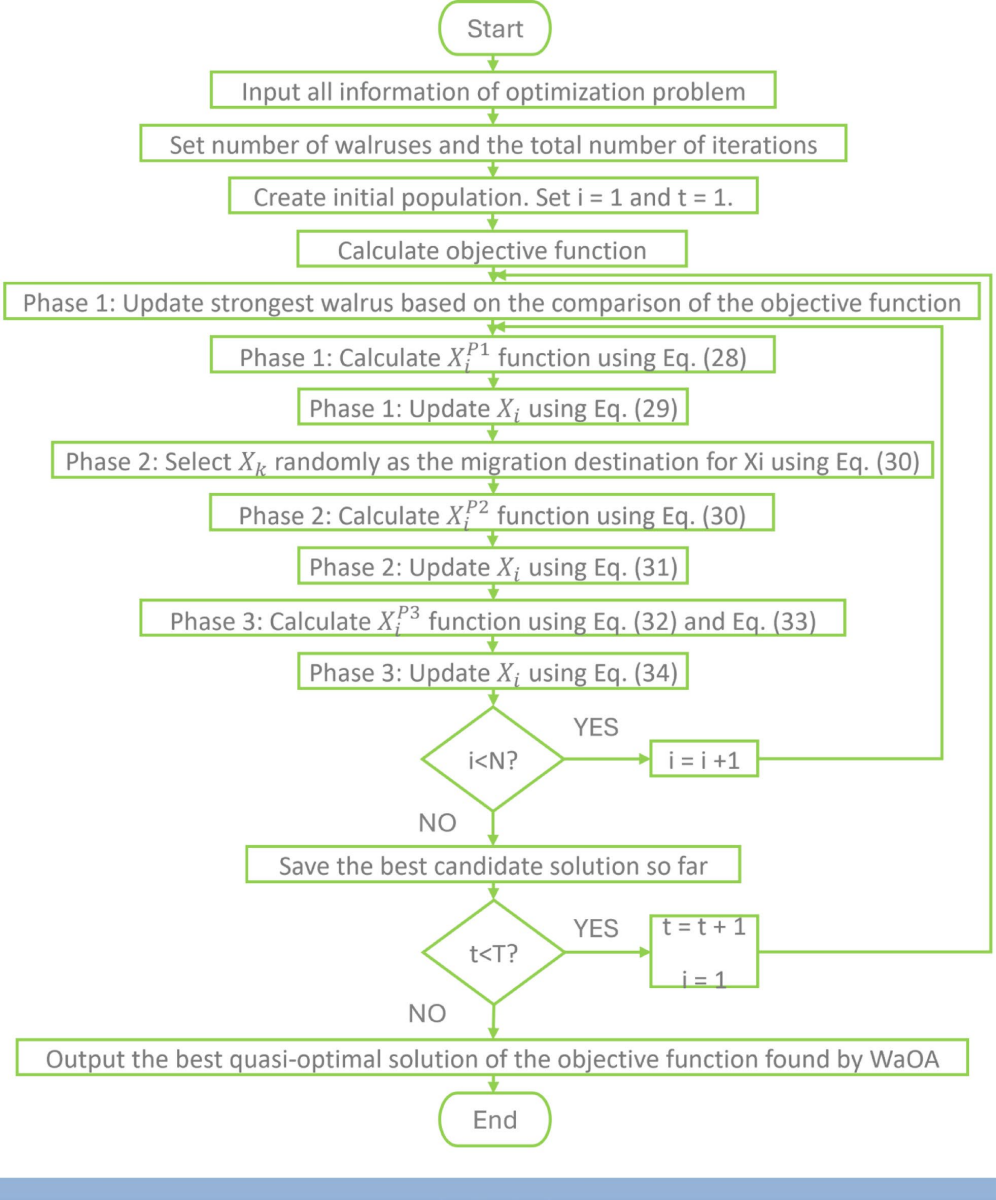
Flowchart of WaOA.

In 2023, the WaOA MATLAB code has been published by M. Dehghani. The equations and parameters on which the code is built are shown in the flowchart in Fig. 15.

These optimization techniques are used to reach the minimum value of relative error, which is the objective function of the optimization technique, by changing the value of A used in the model, determining the range allowed to change the value of A between 80 and 120.

The comparative analysis focuses on relative modeling error, convergence behavior, and dynamic response characteristics under a unified optimization setup, rather than statistical benchmarking across multiple independent runs.

Table 5 displays the results that these optimization techniques have produced.

**Table 5:** Different values of A and the corresponding relative error.

How $A$ is calculated	$A$	Relative error
Curve Fitting	101.2	0.0099
TLBO	92.53460	0.000024373952
CGO	92.51637	0.000000210491
SBOA	92.51648	0.000000110782
WaOA	92.51652	0.000000072613

From the results presented in Table 5, it is clear that the relative error has become much smaller when using optimization techniques compared to its value when using curve fitting, as happened in^12^, and although the relative error does not differ much according to the optimization techniques used, WaOA exhibits the lowest relative error among the investigated techniques and makes the model closer to the lab results. This improvement corresponds to a reduction of more than five orders of magnitude relative to the curve-fitting baseline.

For all investigated optimization techniques, a unified iteration budget of 100 iterations is adopted to ensure a fair comparison. The population size is selected within a standard range (25–50 individuals), consistent with commonly reported values in the optimization literature, and adjusted according to the characteristics of each algorithm. The impact of these parameter choices on optimization performance is reflected in the convergence behavior summarized in Table 6, which reports both convergence iteration and convergence time under an identical stopping criterion.

**Table 6:** Convergence behavior of the investigated optimization algorithms.

Optimization technique	Convergence time (s)	Convergence iteration
TLBO	0.4781	26
CGO	0.4651	26
SBOA	0.4236	26
WaOA	0.3201	24

Convergence is detected for each optimization technique when the relative improvement of the best objective value remained below 1 × 10^−6^ for 20 consecutive iterations. The convergence iteration corresponds to the iteration index at which this criterion is first satisfied, while the convergence time is the elapsed runtime until this point.

From a computational perspective, the reported convergence results indicate that all investigated optimization techniques are cost-effective for SOFC model tuning. Since the algorithms are executed under the same iteration budget, differences in convergence time primarily reflect the computational efficiency of each method rather than differences in stopping criteria or execution limits. These results confirm that the proposed optimization framework remains computationally feasible for offline SOFC modeling and design studies, where accuracy and robustness are prioritized over real-time implementation.

### Operating-condition-dependent performance analysis

There are many factors that affect cell performance and speed of response to changes that occur when operating. These factors may relate to cell manufacturing or operating conditions. With regard to the manufacture of the cell, there are the materials that are used in the manufacture of anode, cathode, and electrolyte, as well as their thickness, the structure of the cell may be supported externally or by anode, cathode, or electrolyte, and there is also the shape of the cell if it is planar or tubular and how gases enter and exit from the cell, the entry of O_2_ and H_2_ may be cross-flow, co-flow or counter flow. In terms of operating conditions, there is the flow rate of gases, the operating temperature, and the magnitude of the change in the electrical load of the cell. In this study, the main concentration will be on the operational aspects of the SOFCs.

#### Conventional SOFC

**Fig. 16:**
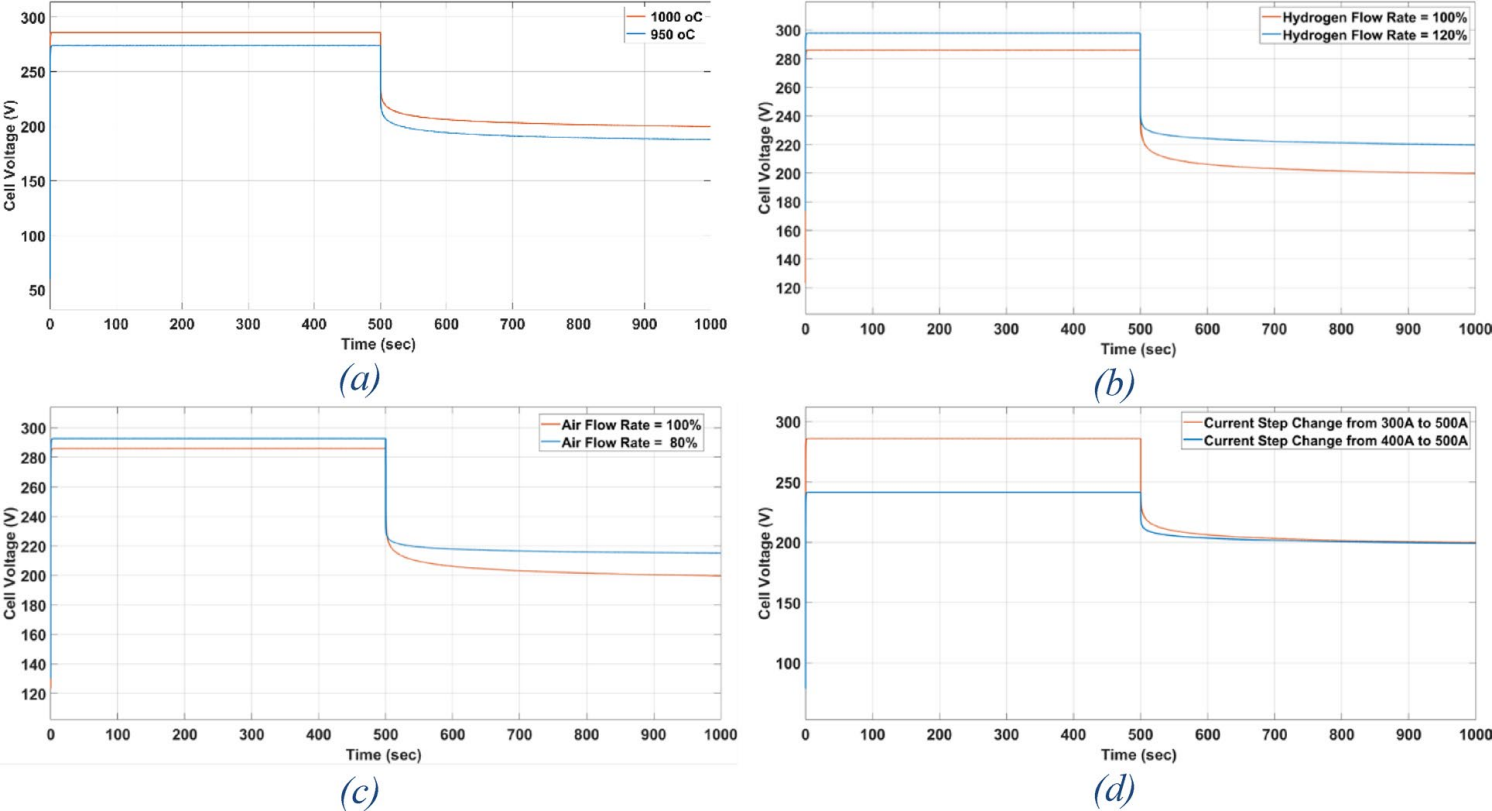
(**a**) The effect of temperature change on time response of conventional SOFC. (**b**) The effect of Hydrogen Flow Rate change on time response of conventional SOFC. (**c**) The effect of Oxygen Flow Rate change on time response of conventional SOFC. (**d**) The effect of Magnitude of change in current on time response of conventional SOFC.

In this part, the effect of changing the operational parameters on the conventional SOFC’s response speed is studied, as shown in Fig. 16^28^.

Temperature adjustments have a negligible influence on the response time of SOFCs, according to the study as shown in Fig. 16a. More specifically, the response time stayed around 460 s in both cases; lowering the temperature from 1000 to 950°C had no noticeable effect on the response speed.

Moreover, a 20% rise in the flow rate is observed to improve the response time when looking at the impact of H_2_ flow rate. In this instance, 460 s at a 100% flow rate reduced to 380 s at a 120% flow rate in response time. That can be shown in Fig. 16b.

Furthermore, reduction in the O_2_ flow rate is associated with faster response time. The response time in this case decreases to 380 s at an 80% flow rate from 460 s at a 100% flow rate as shown in Fig. 16c.

Furthermore, there is a noticeable effect from the magnitude of the variation in electric current. The current change starts off at 300 A and is increased to 500 A. A quicker response is noted when this range is reduced to 400 to 500 A. In the modified range, the response time drops from 460 s in the original range to only 300 s. That can be observed in Fig. 16d.

Table 7 represents the summary of the effect of changes on time response of conventional SOFC explaining that operating temperature has the lowest effect on time response, increasing H_2_ flow rate and decreasing O_2_ flow rate have the same effect on the time response and changing the magnitude of the load Current change has the greatest effect on the time response.

**Table 7:** Summary of the effect of changes on time response of conventional SOFC.

	H_2_ flow rate (%)	O_2_ flow rate	Operating temperature	Current change	Time required to reach the steady state value (s)
Case 1	100	100%	1000 $^\circ {\mathrm{C}}$	300 A $\to$ 500 A	460
Case 2	100	100%	950 $^\circ {\mathrm{C}}$	300 A $\to$ 500 A	460
Case 3	120	100%	1000 $^\circ {\mathrm{C}}$	300 A $\to$ 500 A	380
Case 4	100	80%	1000 $^\circ {\mathrm{C}}$	300 A $\to$ 500 A	380
Case 5	100	100%	1000 $^\circ {\mathrm{C}}$	400 A $\to$ 500 A	300

### Effect of gases flow rate on output power

From the previous results of different cases, it can be noticed that the effect of the change in the flow rate of H_2_ and O_2_ is similar on the speed of the cell response, which led to the study directing the impact of their change on the amount of electrical power generated. Based on that, the WaOA that was previously used in this study will be used to reach the optimal values of the flow rate of the gases that give the highest value of the generated power while maintaining the fuel utilization (U_f_) in the permissible range, which is from 0.7 to 0.9.

U_f_ is the ratio between the amount of H_2_ that is actually consumed inside the cell and the amount of H_2_ entering the cell. The range is determined for the change of this factor between 0.7 and 0.9 because if the value of this factor is less than 0.7, it means that a larger amount than what the cell needs has been entered, and it will exit the cell later as exhaust. It also leads to an increase in the electrical voltage of the cell quickly, increasing the value of concentration losses according to the V-I polarization curve that has been studied previously. And if the value of this factor exceeds 0.9, it means that the cell will suffer from Severe fuel shortages, which lead to rapid cell destruction or at least shortening the life of the cell^14^.

Regarding the operating limitations, the H_2_ flow rate is permitted to vary from 30 to 133% of its rated value, while the O_2_ flow rate is permitted to vary between 80 and 120% of the rated value as shown in Table 8^28^.

**Table 8:** Limits and Conditions used in WaOA to optimize the output power.

	min (%)	max (%)
Hydrogen flow rate	30	133
Air flow rate	80	120

0.7 < U_f_ < 0.9

The model under study results in optimal flow rates of 111.11% for O_2_ and 133% for H_2_ when WaOA is applied. With these parameters, an output power of **91.245** kW is produced at a voltage of 304.15 V, and a current of 300 A.

It is clear how successful WaOA is when comparing the outcomes obtained with those listed in Table 7. Specifically:

- Case 1, with H_2_ flow rate of 100% and O_2_ flow rate of 100%, produced a voltage of 286 V when the current is 300 A and an output power of **85.8** kW.
- Case 3, with H_2_ flow rate of 120% and O_2_ flow rate of 100%, produced a voltage of 298 V when the current is 300 A and an output power of **89.4** kW.
- Case 4, with H_2_ flow rate of 100% and O_2_ flow rate of 80%, resulted in a voltage of 292 V when the current is 300 A and an output power of **87.6** kW.

So, although the flow rates of gases have the same effect in terms of the response speed of the cell, the flow rate of H_2_ has a greater effect in relation to output power.

#### Reversible SOFC

In this part, the effect of changing the operational parameters on the rSOFC’s response speed is studied. All previous studies in literature related to this part are concerned with the changes that occur within the cell itself, such as the temperature gradient or the voltage gradient along the cell, where the attention is focused on the chemical aspect, cell manufacturing, and material development^37^, however, in this study, the focus will be on the electrical aspect and the effect of the changes on the electric current and voltage of the cell, and this is what matters when the cell is connected to the electrical grid later as a generator.

**Fig. 17:**
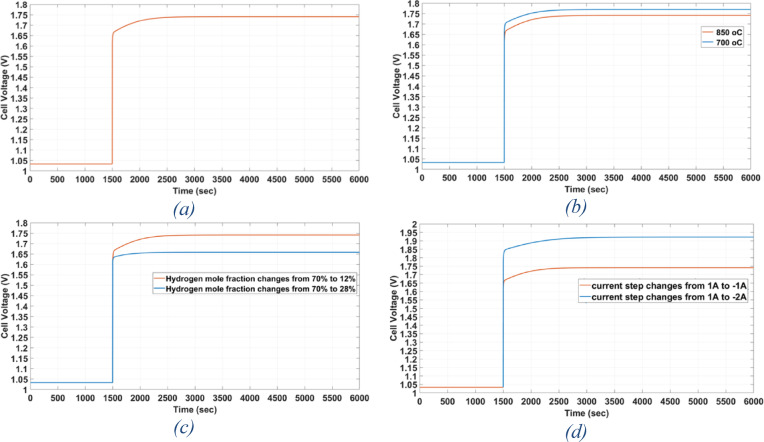
(**a**) dynamic response of rSOFC without any change in the operational conditions. (**b**) The effect of temperature change on time response of rSOFC. (**c**) The effect of Hydrogen mole fraction change on time response of rSOFC. (**d**) The effect of current step change on time response of rSOFC.

For this purpose, the operating conditions mentioned in Table 4, which describe the boundary conditions before and after the mode switching, will be changed. First, the operating temperature in the case of electroyser mode will be changed from 850 to 700°C to look at the effect of thermal dynamics on cell response speed. Secondly, the size of the change in the electric current will be changed to −2 amperes in the case of electroyser mode instead of −1 amperes, as this change is expected to affect the electrochemical reactions that occur in the cell and thus the speed of the cell’s response. Third, the H_2_ mole fraction in the case of electroyser mode will be changed from 12 to 28% to study the effect of the amount of fuel consumed in chemical reactions that take place inside the cell on the speed of its response. Figure 17 shows the relationship between rSOFC voltage and time during mode switching.

The response rate is found to remain relatively constant when the temperature in the electrolyser mode is changed from 850 to 700 °C, as shown in Fig. 17b. It has been found that throughout a period of 31 min, the voltage changes from 1.0329 to 1.7697 V.

When the hydrogen mole fraction is changed, it is found that the voltage has reached 1.6584 V in 1448 s (24 min) with a 28 mol fraction of hydrogen and a 72 mol fraction of water, which is shorter time than a 12 mol fraction of hydrogen and an 88 mol fraction of water, as shown in Fig. 17c.

When the current in the electroyser mode is set to −2 A, it is noted that the voltage reaches 1.9223 V in 2154 s (36 min)—a greater duration than in the initial case—in the situation where the current step is changed, as shown in Fig. 17d.

Table 9 represents the summary of the effect of changes on time response of rSOFC explaining that operating temperature has the lowest effect on time response, changing H_2_ flow rate has a good effect on the time response and changing the magnitude of the Current change has the greatest effect on the time response.

**Table 9:** Summary of changes on time response during mode switching of rSOFC.

	Hydrogen mole fraction	Operating temperature	Current change	Time required to reach the steady state value (s)
Case 1	70% $\to$ 12%	600 $^\circ {\mathrm{C}}$ $\to$ 850 $^\circ {\mathrm{C}}$	1 A $\to$ −1 A	1844
Case 2	70% $\to$ 12%	600 $^\circ {\mathrm{C}}$ $\to$ 700 $^\circ {\mathrm{C}}$	1 A $\to$ −1 A	1844
Case 3	70% $\to$ 28%	600 $^\circ {\mathrm{C}}$ $\to$ 850 $^\circ {\mathrm{C}}$	1 A $\to$ −1 A	1448
Case 4	70% $\to$ 12%	600 $^\circ {\mathrm{C}}$ $\to$ 850 $^\circ {\mathrm{C}}$	1 A $\to$ −2 A	2154

## Conclusion

This paper uses simulation to study both static and dynamic models for conventional and reversible SOFCs.

The impact of temperature and flow rate on the voltage-current relationship for SOFCs is highlighted by the analysis of the static model. It is discovered that for conventional SOFCs, voltage is improved at a given current by raising the temperature or flow rate. On the other hand, increasing temperature causes a drop in the electrolyser mode voltage of reversible SOFCs while increasing the fuel cell mode voltage.

To improve the static model accuracy and make its results closer to the published experimental results, various optimization techniques such as TLBO, CGO, SBOA and WaOA have been used, and WaOA exhibited the lowest relative error among the investigated techniques under the studied conditions. It has provided the most accurate and reliable results, closely matching the experimental results.

The dynamic model states that it takes minutes for voltage to stabilize after the electrical current modification. For both types of cells, temperature changes have very little effect on how long it takes for the voltage to stabilize. The amount of the current change has a significant impact on response time; larger changes need longer time for voltage stabilization. The study also has looked at the impact of flow rate on response speed and found that, for conventional SOFCs, higher hydrogen and lower oxygen flow rates lead to better performance, and that, for reversible SOFCs, higher hydrogen flow rates in the electrolyser mode speed up stabilization after mode switching from fuel cell mode to electrolyser mode.

It is also concluded that increasing the hydrogen flow rate and decreasing oxygen flow rate result in higher voltage for the same current so higher output power, but the effect of hydrogen flow rate is more noticeable.

From an engineering perspective, the proposed optimization-based SOFC modeling framework provides a practical tool for system-level design and operation optimization, while also enabling the prediction of fuel cell behavior in grid-connected applications. By supporting accurate model tuning and operating-condition-dependent analysis, the framework allows informed decisions related to reactant flow-rate adjustment, operating temperature selection, and performance enhancement during the design stage. In addition, the accurate characterization of voltage stabilization behavior following current variations enables the assessment of SOFC dynamic response under load changes and network-induced operating fluctuations, which is particularly relevant for grid-connected SOFC systems.

This conclusion can stimulate the improvement of fuel cell materials to withstand operating conditions that enable enhanced performance, which in turn supports improved SOFC operation when connected to electrical networks.

## Data Availability

All data generated or analysed during this study are included in this published article. For any additional clarification, please contact the corresponding author.
